# Acute cigarette smoke exposure activates apoptotic and inflammatory programs but a second stimulus is required to induce epithelial to mesenchymal transition in COPD epithelium

**DOI:** 10.1186/s12931-017-0565-2

**Published:** 2017-05-03

**Authors:** Lynne A. Murray, Rebecca Dunmore, Ana Camelo, Carla A. Da Silva, Malin J. Gustavsson, David M. Habiel, Tillie L Hackett, Cory M. Hogaboam, Matthew A. Sleeman, Darryl A. Knight

**Affiliations:** 1 Respiratory, Inflammation and Autoimmunity, MedImmune Ltd, Granta Park, Cambridge, CB21 6GH United Kingdom; 2 Respiratory, Inflammation and Autoimmunity innovative Medicines Unit, AstraZeneca R&D, Mölndal, Sweden; 3 Department of Anesthesiology, Pharmacology and Therapeutics, University of British Columbia, Vancouver, Canada; 4 Department of Medicine, Cedars-Sinai Medical Center, Los Angeles, CA USA; 5 James Hogg Research Centre, University of British Columbia, Vancouver, Canada; 6 School of Biomedical Sciences and Pharmacy, Newcastle, Australia; 7 Priority Research Centre for Healthy Lungs, University of Newcastle and Hunter Medical Research Institute, New South Wales, Australia

**Keywords:** TGFβ1, Poly I:C, Remodelling, Apoptosis

## Abstract

**Background:**

Smoking and aberrant epithelial responses are risk factors for lung cancer as well as chronic obstructive pulmonary disease and idiopathic pulmonary fibrosis. In these conditions, disease progression is associated with epithelial damage and fragility, airway remodelling and sub-epithelial fibrosis. The aim of this study was to assess the acute effects of cigarette smoke on epithelial cell phenotype and pro-fibrotic responses* in vitro* and* in vivo*.

**Results:**

Apoptosis was significantly greater in unstimulated cells from COPD patients compared to control, but proliferation and CXCL8 release were not different. Cigarette smoke dose-dependently induced apoptosis, proliferation and CXCL8 release with normal epithelial cells being more responsive than COPD patient derived cells. Cigarette smoke did not induce epithelial-mesenchymal transition. In vivo, cigarette smoke exposure promoted epithelial apoptosis and proliferation. Moreover, mimicking a virus-induced exacerbation by exposing to mice to poly I:C, exaggerated the inflammatory responses, whereas expression of remodelling genes was similar in both.

**Conclusions:**

Collectively, these data indicate that cigarette smoke promotes epithelial cell activation and hyperplasia, but a secondary stimulus is required for the remodelling phenotype associated with COPD.

**Electronic supplementary material:**

The online version of this article (doi:10.1186/s12931-017-0565-2) contains supplementary material, which is available to authorized users.

## Background

Chronic obstructive pulmonary disease (COPD) is a chronic lung disease commonly associated with inhalation of cigarette smoke (CS). While the pathology of COPD is generally considered to be destructive in nature, epithelial remodeling and sub-epithelial fibrosis of the small airways is now recognized as a key histopathological feature of the disease [1, 2]. While CS directly activates and damages the epithelium, viral infections, which occur frequently in smokers with and without COPD also influences epithelial phenotype and function [3]. Moreover, viral-induced exacerbations contribute significantly to disease progression, accelerated decline of lung function and disease morbidity and mortality [3]. Unfortunately, the mechanisms that drive changes to the epithelium in COPD following exposure to CSE and respiratory viruses remains largely unexplored.

What is known, suggests that damage to the epithelium triggers a temporal cascade of inflammatory and cell signaling events that under normal circumstances leads to inflammation, resolution of inflammation and repair. However, under conditions of chronic inflammation, the epithelium fails to repair effectively with structural and functional changes including goblet cell hyperplasia, squamous cell metaplasia, induction of pro-inflammatory chemokines [4], matrix metalloproteinases [5] and epithelial cell apoptosis and proliferation [6]. The mechanisms by which these changes occur are unclear, although endoplasmic reticulum (ER) stress and oxidative damage have been proposed as initiators of epithelial cell apoptosis in COPD [7–11].

One aberrant epithelial response that has been associated with abnormal repair and fibrosis is epithelial to mesenchymal transition (EMT). This phenomenon, commonly observed in cancer and a component of normal organ development, occurs when epithelial cells transform into highly motile mesenchymal cells. EMT has been shown to contribute to airway disease and fibrosis in several organs, including the lung [12, 13]. However, the underlying mechanisms and the functional consequences of EMT in the airways from COPD patients remain unclear [14].

In this study we investigated the epithelial phenotype in COPD and responses to CS extract (CSE) on normal and COPD bronchial epithelial cell function in vitro. We assessed the impact of CSE on apoptosis, proliferation and chemokine production. We show that in the short term, cigarette smoke does not modulate EMT, but rather induces epithelial cell proliferation, apoptosis and CXCL8/IL8 production. Exposing mice to CSE combined with poly I:C challenge revealed that CSE induces apoptosis and inflammation in the lung, but a secondary stimulus of poly I:C amplifies fibrotic changes. These data suggest that CS alone is insufficient to induce the chronic airway remodeling that is observed in COPD and that an extra insult, such as viral infection is required.

## Methods

### Human COPD cohort

This study was approved by the Research Ethics Board of the University of British Columbia, Canada. Human lung tissue from patients with COPD (*n* = 37) and controls (*n* = 9) was obtained from patients undergoing surgery and who gave informed consent. All COPD patients were GOLD II/III, the majority were male and ex-smokers undergoing surgery for lung cancer. Control lung tissue was from otherwise healthy, non-smoking subjects whose lungs were not used for transplant from the IIAM as previously described (Hackett et al., [12]). We attempted to age match as best as possible, but the normal donors were significantly younger than the COPD cohort.

### Epithelial cell culture

Normal human bronchial epithelial (NHBE, donor 105116, donor 7 F4334 or 84749) cells and COPD human bronchial epithelial cells (COPD-AEC donor OF3207, Lonza, UK) were maintained in BEGM™ (bronchial epithelial cell growth medium) plus BulletKit™ (Lonza). Both cell types were cultured at 37 °C in the presence of 5% CO_2_. Primary bronchial epithelial cells were also isolated from patients and cultured as previously described [12]. In all in vitro epithelial cell experiments, control and COPD epithelial cells were used before passage 4. Cells were seeded for each experiment so they would be between 70 and 80% confluent at the start of each experiment. In addition, cells were deprived of bovine pituitary extract (BPE), hydrocortisone, human epidermal growth factor (EGF), epinephrine, transferrin and triiodothyronine for 16 h prior to stimulations. Insulin, retinoic acid and GA-1000 remained throughout the experiment.

### Induction of EMT

Monolayer cultures were grown to 60–70% confluence on 6-well tissue culture plates (BD Falcon, NJ), before being quiesced and exposed to TGFβ1 (10 ng/ml) for 48 h. Samples were then assayed for mesenchymal (EDA-Fn) and epithelial (E-Cadherin) markers using quantitative polymerase chain reaction and immunoblot.

### Cigarette smoke extract (CSE) production

Cigarette smoke extract (CSE) 100% stock was generated from mainstream cigarette smoke by the combustion of 1 1R3F reference cigarette (Tobacco Health Research, University of Kentucky, Kentucky, USA) with the filter removed, using a peristaltic pump, through 25 mls of culture medium without FBS. The CSE was then sterile filtered through a 0.22 μM filter, and added to cell cultures at the required concentration.

### Immunohistochemical staining of human airway sections

Tissue sections were deparaffinized and rehydrated, and put through antigen retrieval by autoclaving (15 min, 120 C, 30 psi) for 20 min in citrate target retrieval solution (Dako, Mississauga, ON, Canada). Subsequently, endogenous peroxidase was quenched with 3% H2O2 and non-specific interactions blocked for 30 min with 10% goat serum. Antibodies directed against α-smooth muscle actin (ab5694, Abcam, Cambridge, MA), and vimentin (AF2105, R&D Systems) were added overnight at 4 °C in 25% goat serum. Sections were then incubated with either biotinylated goat anti-mouse or goat anti-rabbit secondary antibody (1:100, Vector Labs, Burlingame, CA) for 60 min followed by a 10-min treatment with Streptavidin-HRP (Dako). The antigen of interest was visualized by 3,3-diaminobenzidine (Dako) and counterstained with Harris Hematoxylin Solution (Sigma, Oakville, ON, Canada). Sections were then dehydrated and mounted with Cytoseal 60 (Richard-Allan Scientific, Kalamazoo, MI). Antibody dilutions and all washes were in TRIS-buffered saline solution.

### Gene expression analyses

For gene expression analysis from lung tissue, total RNA was obtained using TRIzol reagents (Invitrogen, Paisley, UK) according to manufacturer’s instructions. RNA was reverse transcribed into cDNA using the high capacity cDNA kit (Applied Biosystems) and gene expression levels were determined using qRT-PCR using an ABI Prism 7900HT sequence detector (Applied Biosystems). Transcript levels of genes of interest were normalised to 18s and fold change was to matched animal controls or non-COPD lung using 2^-ΔΔCt^.

### Nu-PAGE and Western blotting

NHBE cells were seeded at 12 × 10^4^ cells/ml and cultured for 24 h in complete medium. Prior to stimulation cells were cultured overnight in basal medium supplemented with only retinoic acid, insulin and antibiotics. TGFβ_1_ (10 ng/ml, R&D Systems, Abingdon, UK), CSE (5% or 10%), or both combined were added to the cells (media alone was used as a control) and left to incubate for a further 48 h. Cells were scraped and lysed in RIPA buffer (Sigma-Aldrich, Poole, UK) containing protease inhibitors (Roche Diagnostics, East Sussex, UK). Cell suspensions were cleared by centrifugation at 8000 x *g* for 10 min at 4 °C. 15 μg of protein was loaded onto a Bis-Tris (4–12%) Nu-PAGE gel (Invitrogen, Paisley UK) followed by electrophoresis and then transferred to PVDF membranes using the iBlot dry blotting system (Invitrogen, Paisley, UK). Membranes were blocked in Odyssey blocking buffer (LI-COR) for 2 h and then probed with E-cadherin (clone 67A4 mIgG1, Santa Cruz, Heidelberg, Germany), fibronectin (clone IST-9 mIgG1, Santa Cruz, Heidelberg, Germany) or beta tubulin loading control (LI-COR) diluted in Odyssey blocking buffer (LI-COR) supplemented with 0.02% Tween-20 overnight at 4 °C. Membranes were washed 4 times in PBS-T for 5 min each and were then probed with anti-mouse or anti-rabbit IRDye 800cw conjugated secondary antibody (LI-COR) in Odyssey blocking buffer supplemented with 0.02% Tween-20. Membranes were washed as before and left to dry before scanning on the Odyssey Clx Infrared Imaging System (LI-COR). Western blots were analysed by densitometry using loading control for normalisation and the fold change in expression was determined compared to untreated controls. Experiments were performed on 3 NHBE donors.

### CXCL8 ELISA

CXCL8 was measured from the supernatants of epithelial cells using a duoset ELISA (R&D Systems) according to manufacturer’s guidelines. Experiments were done three times using triplicate wells each time.

### Apoptosis assay

NHBE and COPD-AEC were plated at 1x10^5^cells/ml and cultured for 24 h to reach around 80–90% confluency. BEGM media with 5, 10 or 20% CSE was added to cells for 6 and 24 h. Caspase-3/-7 activity was determined from treated epithelial cells using the Caspase-Glo® -3/-7 Assay (Promega) according to manufacturer’s guidelines. Experiments were done three times using triplicate wells each time.

### Lactate Dehydrogenase (LDH) assay

To determine the levels of released LDH in cell-free supernatants, the CytoTox 96 Non-Radioactive Cytotoxicity Assay (Promega) was used to measure levels in the supernatants of epithelial cells according to manufacturer’s guidelines. Measurements were performed on three individual experiments using triplicate wells each time.

### Proliferation assay

NHBE and COPD-AEC cells were plated at 1x10^5^cells/ml and cultured to 80–90% confluency. At this point, cells were exposed to CSE media at a concentration of 5, 10 and 20% in low serum conditions. Cells were stimulated with media alone, CSE media, full serum complete media or media with 30 ng/ml of PDGF (R&D systems, UK) as a positive control for proliferation for 72 h. At this point cells were pulsed with 30 μl/ml of [3^H^] thymidine for 6 h and proliferation measured in a topcount liquid scintillation counter (Perkin-Elmer). Results were expressed as counts per minute. Experiments were done three times using triplicate wells each time.

### Mice

Six-week-old C57Bl/6 mice were purchased from Taconic Europe (Denmark). Experiments were conducted in accordance with standards established by the Council of Europe, AstraZeneca Global R&D Standards for animal care, European Union Directive 86/609, and Swedish legislation. The studies were approved by the regional Ethics Board, Malmö/Lund, Sweden (reference number M230/09).

### Animal exposure to cigarette smoke and poly I:C

The current protocol was adapted from a previously described model by Kang et al. [16]. Briefly, mice were exposed to cigarette smoke using a whole-body smoke exposure system (SIU-48, Promech Lab AB, Vintrie, Sweden). Mice were exposed to 10 1R3F reference cigarettes with filters removed for a period of approximately 50 min, twice daily, for 18 consecutive days, control animals (i.e., sham-exposed animals) were exposed to room air only. Prior to exposure mice were acclimatized over a 3 day period. On days 8, 11, 15 and 18 of cigarette smoke exposure, mice were lightly anesthetized and 50 μl aliquots of poly (I:C) (50 μg per animal, Sigma Aldrich, Sweden) or its vehicle control were administered via nasal aspiration. Animals were euthanized at day 19 of the protocol and outcome measures assessed.

### Evaluation of airway inflammation and remodelling in mice

Tissue samples and bronchoalveolar lavage (BAL) fluid were collected for assessment. For histologic evaluations, the left lung was inflated to 25 cm with 4% buffered formaldehyde (Histolab Products AB, Sweden). Images of lung sections were captured at x20 final magnification on a microscope using a Scanscope (Aperio, CA, USA). In addition, RNA was extracted from tissue for gene expression analysis (see previous section). Total cellularity was determined from BAL fluid and specific blood cell counts were determined for macrophages, neutrophils, lymphocytes and eosinophils.

### Immunohistochemical staining

Immunohistochemical staining for Hematoxylin and eosin (H&E), proliferating cell nuclear antigen (PCNA); Novocastra Laboratories Ltd., Newcastle upon Tyne, UK,) and caspase-3 (rabbit anti-human/mouse caspase-3 active, AF835; R&D Systems, UK) were performed. Antibodies were detected with EnVision™ kit (DAKO UK Ltd, Cambridgeshire) and DAB as chromogen, according to the manufacturer’s instructions.

### Statistical analysis

Normal distribution was assumed. Data were expressed as means ± SEM, and assessed for significance by Student's *t* test or ANOVA as appropriate.

## Results

### Cigarette smoke induces apoptosis, proliferation and chemokine generation in normal and COPD lung epithelial cells

**Fig. 1:**
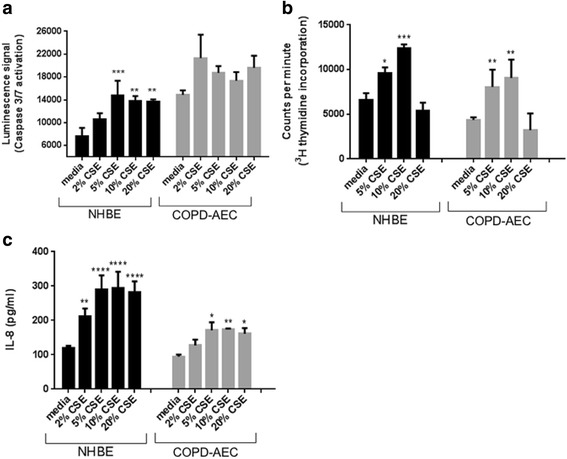
Cigarette smoked induces apoptosis, proliferation and IL-8 production in normal and COPD epithelial cells. NHBEs (normal human bronchial epithelial cells) or COPD-AECs (COPD diseased human bronchial epithelial cells; Lonza) were treated with increasing concentrations of CSE (2–20% as indicated) and after 24 h CSE promoted apoptosis was increased as assessed by elevated caspase 3/7 activity (**a**), and cell proliferation (**b**) as measured using ^3^H-incorporation, (*n* = 3). CXCL8/IL8 was induced in a dose-dependent manner by CSE treatment (**c**), as measured by ELISA in the supernatants following stimulation for 24 h (*n* = 2 donors, each repeated *n* = 3 times).

To determine the effect of CSE on apoptosis, airway epithelial cells from normal (NHBE) and COPD (COPD-AEC) lung were treated with increasing concentrations of CSE for 6 h or 24 h and caspase 3/7 activity was measured. There was no increase in caspase activity at 6 h (data not shown), however CSE induced an increase in caspase 3/7 activity at 24 h, with caspase activity maximal at a concentration of 5% CSE (Fig. 1a). Baseline caspase 3/7 activity was significantly higher in COPD-AEC compared to NHBE and CSE had only a modest stimulating effect, suggesting that endogenous caspase activity was already near maximum at rest (Fig. 1a). The concentrations of CSE used did not affect epithelial cell viability as observed morphologically (Additional file 1: Figure S1) or when assessing lactate dehydrogenase release (Additional file 1: Figure S2).

Next we assessed the impact of CSE on epithelial cell proliferation and chemokine production. CSE increased proliferation of NHBE and COPD-AEC at 5% CSE and 10% CSE (Fig. 1b). We also measured the effects of CSE on the release of CXCL8, a chemokine known to be induced by CS [15], and found that CSE induced CXCL8 release in a concentration dependent manner from both NHBE and COPD-AEC, although surprisingly IL-8 release from COPD-AEC was blunted in response to CSE when compared to the NHBE (Fig. 1c).

### Acute cigarette smoke exposure does not affect epithelial to mesenchymal transition

**Fig. 2:**
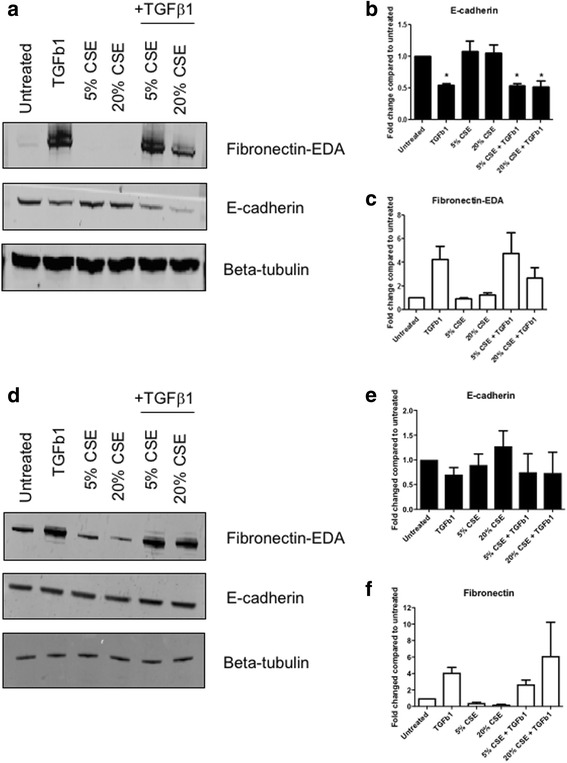
TGFβ1 but not CSE drives a mesenchymal phenotype in lung epithelial cells. Exposure of NHBE (**a**-**c**) or COPD-AEC (D-F) cells to TGF β1 (10 ng/ml) decreased E-cadherin and increased EDA-Fn expression (A-C). Exposure to CSE (5%) did not impact on E-cadherin expression in the presence or absence of TGF-β1 (**a**, **b**), but at a high concentration (20%) did reduce TGFβ1-induced EDA-Fn expression (**a**, **c**). COPD-AEC were resistant to EMT. Exposure of AEC from COPD patients (Lonza) to TGFβ1 had minimal effect on E-Cadherin expression (**d**, **e**) although it did induce EDA-Fn expression (**d**, **f**). Exposure to CSE (5%) did not impact E-Cadherin (**d**, **e**) or EDA-Fn (**d**, **f**) expression in the presence or absence of TGF-β1. Representative western blots from NHBE donor cells (**a**) or COPD donor cells (**d**), shown alongside densitometry from 3 NHBE donors (**b**, **c**) or 1 COPD donor repeated 3 times (**e**, **f**), protein levels normalised to β-tubulin loading control

We next assessed the effects of CSE on markers of EMT. Epithelial cells from non-diseased donors were stimulated for 48 h with CSE in the presence or absence of 10 ng/ml TGFβ1 and EMT markers such as e-cadherin and the EDA splice variant of fibronectin were measured. As shown in Fig. 2, TGFβ1 reduced E-cadherin expression and concomitantly increased expression of EDA-Fn suggesting an EMT response. In contrast, addition of CSE at either 5 or 20% did not directly induce EMT nor modulate TGFβ1-induced EMT (Fig. 2a-c).

### Decreased epithelial markers and elevated mesenchymal markers in COPD

**Fig. 3:**
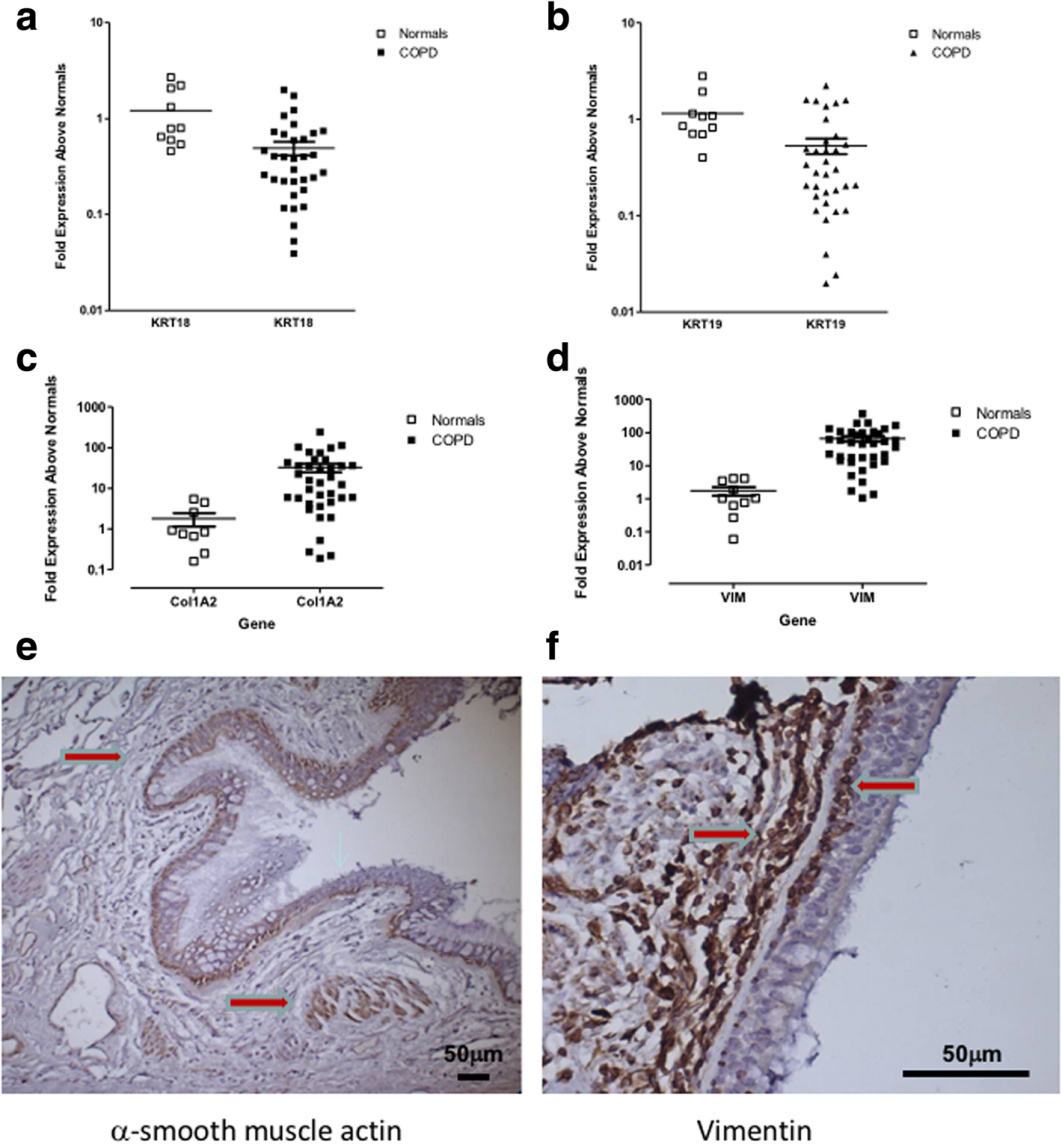
Epithelial and mesenchymal marker expression in COPD lung. Lung tissue samples were analysed for epithelial marker gene expression (**a**) *krt18* and (**b**) *krt19* which were down regulated in patients with COPD whilst the mesenchymal markers (**c**) *col1a1* and (**d**) *vim* were concomitantly upregulated. Immunohistochemical staining of COPD lung sections identified positive staining (*brown*) for (**e**) alpha-smooth muscle actin and (**f**) vimentin, which appeared to be localised to the epithelial layer as indicated by the arrows

To then determine if any quantitative differences in epithelial and mesenchymal markers exist in the COPD lung, diseased and healthy lung biopsy tissue was assessed for the gene expression of epithelial and mesenchymal specific cell markers. As shown in Fig. 3a-d, expression of the epithelial specific markers *keratin-18* and *keratin-19* were downregulated in COPD lung tissue, whilst the expression of mesenchymal markers *collagen 1a2* and *vimentin* were highly upregulated compared to control lung tissue (Fig. 3a-d). To further support these findings and localise the increase in mesenchymal markers, immunohistochemical staining identified alpha-smooth muscle actin (αSMA) and vimentin staining within the epithelial layer itself (Fig. 3e).

### Cigarette smoke induces epithelial cell apoptosis *in vivo* but fibrotic changes only occur after viral exacerbation

**Fig. 4:**
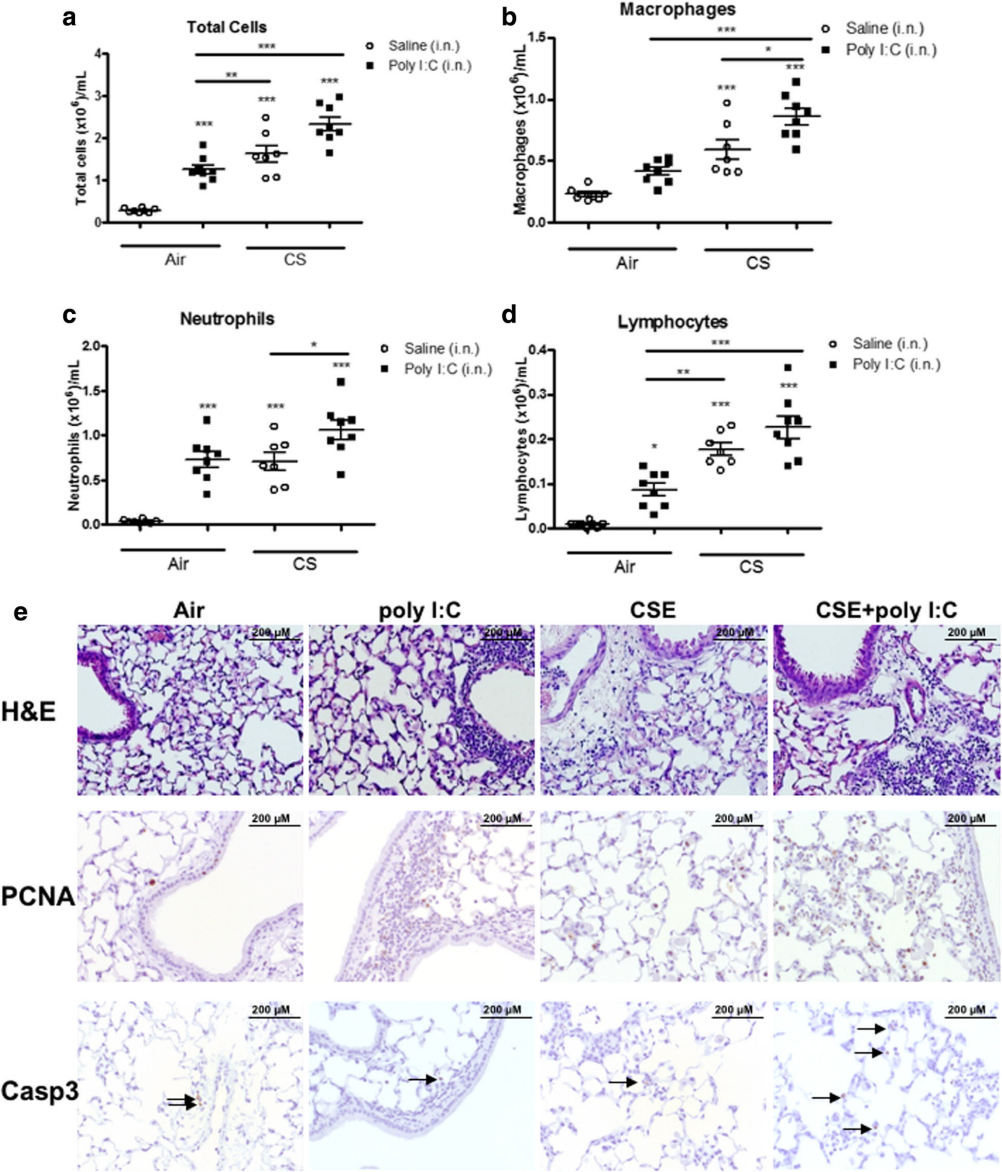
Cigarette smoke-induced lung inflammation is moderately enhanced by poly (I:C) in mice. C57Bl/6 mice were exposed to cigarette smoke (CSE), poly (I:C) (pI:C, 50 μg), a combination of both (CSE + pI:C) or their respective controls (Air). One day after the last poly (I:C) challenge (Day 19 of the protocol), the mice were killed, bronchoalveolar lavage (BAL) was undertaken, and lung sections were prepared. CSE induced an increase in BAL total cell number (**a**), neutrophil (**b**), macrophage (**c**) and lymphocyte (**d**) number. **e** Histological assessments indicated an increase in interstitial inflammation (hematoxylin & eosin; H&E; top panel) and apoptosis (proliferating cell nuclear antigen or cyclin; PCNA; middle panel stains, caspase-3; casp3, bottom panel stains, x20 of original magnification) following CSE and poly I:C challenge. Results are presented as the mean ± SEM of 6–8 animals per group. * *p* < 0.05, ** *p* < 0.01, *** *p* < 0.005

**Fig. 5:**
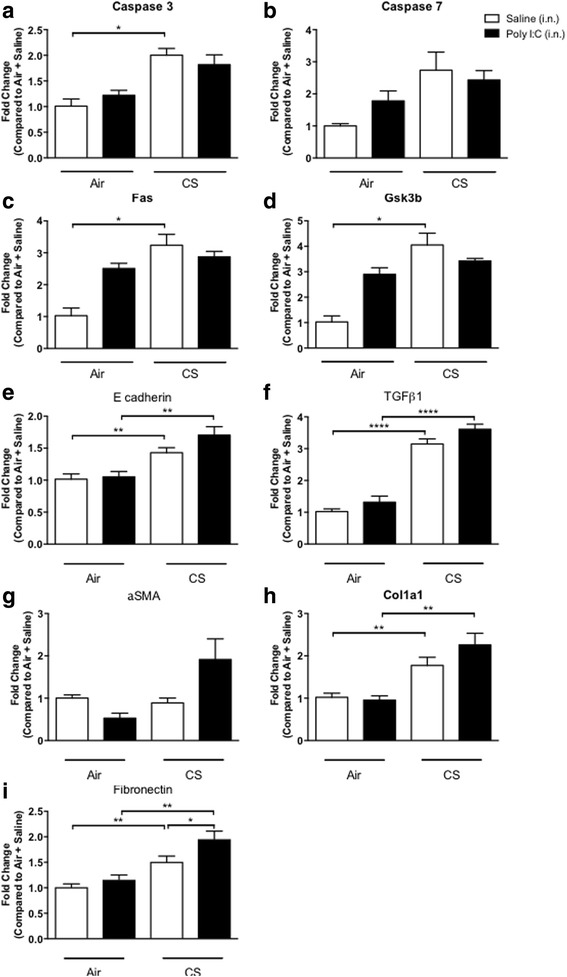
Cigarette smoke alone induces apoptotic markers in the lungs of mice and increases some mesenchymal marker expression although this is further enhanced by poly (I:C). C57Bl/6 mice were exposed to cigarette smoke (CSE), poly (I:C) (pI:C, 50 μg), a combination of both (CSE + pI:C) or saline controls. One day after the last poly (I:C) challenge, the mice were killed and lung tissue was taken for RNA extraction and gene transcript analysis. The apoptotic markers *caspase 3* (**a**)*, caspase 7* (**b**)*, fas* (**c**) and *gsk3b* (**d**) were increased by cigarette smoke alone and no further enhancement was observed in combination with poly (I:C). The epithelial marker *e-cadherin* (**e**) was slightly elevated in the presence of cigarette smoke. *tgfβ1* (**f**) expression was clearly upregulated following cigarette smoke exposure and further still when in combination with poly I:C. The mesenchymal markers *alpha smooth muscle actin* (**g**), *collagen1a1* (**h**) and fibronectin (**i**) were also more robustly increased in the combined poly I:C cigarette smoke group. * *p* < 0.05, ** *p* < 0.01, **** *p* < 0.001

We next used an in vivo mouse model of virus-induced COPD exacerbations using cigarette smoke with poly I:C [16]. Exposure to CSE or poly I:C challenges significantly induced an inflammatory response, as observed by increased infiltration of macrophages, neutrophils and eosinophils in the bronchoalveolar lavage (BAL) of animals compared to animals exposed to air alone. In addition, further enhancement of the inflammatory effect was observed when CSE and poly I:C were used in combination (Fig. 4a-d). Histological analysis of lung sections showed PCNA staining in areas of inflammation in the lungs of animals treated with CSE or poly I:C and this was further enhanced in mononuclear cells and type II pneumocytes when CSE and poly I:C were administered in combination (Fig. 4e). Minimal caspase-3 staining was observed in control animals, while a marginal staining in the perivascular inflammatory cuffs was observed in the poly I:C group. Exposure to CSE significantly increased caspase-3 staining, primarily in the areas of alveolar inflammation (Fig. 4e). Addition of poly I:C had no effect on the CSE induce caspase-3 expression. In addition to increased expression of caspase 3 and -7 (Fig. 5a and b), we also observed increases in other apoptotic markers such as *fas* (Fig. 5c) and *gsk3b* (Fig. 5d) with CSE alone or in combination with poly I:C. In contrast, we saw an increase in the epithelial marker *E-cadherin* (Fig. 5e), the mesenchymal markers *tgfb1* (Fig. 5f), *aSMA* (Fig. 5g), *collagen 1a1* (Fig. 5h) and *fibronectin* (Fig. 5i), but the most robust and elevated responses were observed in the animals treated with CSE and poly I:C. This suggests that an additional stimulus, beyond CSE, is required for abnormal repair and fibrosis to occur.

## Discussion

Given its prime location, it is perhaps not unexpected that the epithelium of people who smoke, and particularly those with COPD would be abnormal. Indeed, chronic inflammation and remodeling of the mucosa are typical features of COPD. We were interested in looking at the effects of CSE on the epithelium and found that using primary epithelial cells from patients with COPD and healthy controls: (1) COPD-AEC have increased basal levels of apoptosis and that exposure to CSE increases caspase activity in healthy and COPD-AEC; (2) CSE increases proliferation and IL-8 release in a concentration-dependent manner although intriguingly, IL-8 release by COPD-AEC was significantly lower than normal-AEC. (3) We then used a mouse model of CSE and poly I:C to mimic viral exacerbations and show that while poly I:C clearly induces lung inflammation in its own right and enhances CSE-induced inflammation, it has no effect on caspase-3/-7 activity either alone or in combination with CSE; (4) finally we show that short-term CSE challenge has no effect on markers of EMT either *in vitro* or *in vivo*. Our data suggests that cigarette smoke induces a marked apoptotic burden on epithelial cells, but at the concentrations and times used does not induce EMT either *in vitro* or *in vivo*.

One of the strengths of our study was the use of primary AEC from COPD patients where we show for the first time that basal apoptotic load is significantly higher in cultured AEC obtained from these patients than AEC obtained from healthy donors. While caspase activity increased in AEC from normal donors after CSE exposure, we saw no statistically significant effect in COPD-AEC. However, basal caspase activity in COPD-AEC was the same as the maximal CSE-induced activity in normal-AEC, suggesting activity of caspase -3/7 is constitutively high in COPD-AEC. In contrast, Chiappara and colleagues [17] showed that epithelial caspase-3 expression was similar in patients with COPD and normal controls and that CSE did not influence apoptosis in a transformed human airway epithelial cell line (16HBE). We hypothesize that since Chiappara et al., examined expression but not activity of caspase-3 and used a transformed cell line which are generally resistant to apoptosis are possible reasons for these disparate findings. We also investigated caspase-3 expression as well as caspase-3/7 activity in a mouse model of CSE and poly I:C to mimic viral exacerbations. We show that poly I:C clearly induces lung inflammation and enhances CSE-induced inflammation, that it increases caspase-3 expression in areas of alveolar inflammation but has no effect on lung tissue caspase-3/7 activity either alone or in combination with CSE. A similar model developed by Kang *et al* [16] demonstrated that the combination of CSE and poly I:C were associated with the induction of type I interferon (IFN) and IL-18, followed by the induction of IL-12/IL-23 p40 and IFN-γ, and the activation of PKR (double-stranded RNA-dependent protein kinase) and eIF-2α (eukaryotic initiation factor-2α). These authors also demonstrated that CSE enhanced the effects of influenza, but no other agonists of innate immunity, suggesting that CSE selectively augments the airway and alveolar inflammatory and remodelling responses induced in the murine lung by poly I:C and viruses [16]. Furthermore, Chiappara et al also showed that expression of two different proliferation markers, PCNA and Ki67 were differentially expressed in epithelium in COPD sections; expression of PCNA was virtually absent in COPD and smoking groups, whereas Ki67 expression was significantly higher in the COPD/smoking groups compared to control. Proliferation was not quantified in vitro. Our data suggest that CSE induces a concentration-dependent proliferative response in vitro, which was maximal at 10% and then sharply declining, presumably due to toxicity. CSE also promoted lung epithelial cell proliferation in vivo as observed by PCNA staining. These results are supported by previous reports that showed smoking generates a dose-dependent pro-proliferative response in epithelial cells of smokers [18].

Release of CXCL8 from normal-AEC dose dependently increased with CSE exposure. Kode *et al* showed similar effects in primary cultures of small airway epithelial cells [19]. In agreement with previous studies [20], basal CXCL8 release was similar in normal and COPD-AEC. These findings differ from those of Schneider et al.*,* who observed increased basal CXCL8 production in epithelial cells from COPD patients [21]. These disparate findings may be due the different culture procedures, as Schneider et al generated differentiated air-liquid interface cultures, whereas both Heijink and colleagues and this study used submerged monolayer cultures. Likewise, disease severity may play a role. Although GOLD status was not reported, 8 of the COPD patients included in the study by Schneider had severe emphysema. Nevertheless, Schulz et al [22] were also not able to detect differences in baseline CXCL8 production in submerged cultured PBEC from currently smoking COPD patients and smoking controls.

Epithelial-mesenchymal transition (EMT) has been categorized into three different types: Type I representing a natural process such as occurs during embryogenesis; Type II – leading to organ fibrosis and Type III, pro-angiogenesis and associated with cancer. In the context of COPD, Sohal and colleagues documented Type III EMT in the epithelium of large airways from patients with COPD [23] and Nishioka et al., showed that epithelial cells from COPD patients have display a partial EMT phenotype under basal conditions [24–30]. Recent findings indicate that similar changes can also be observed in the small (<1 mm) airways of smokers. However, despite CSE-induced increases in expression of TGFβ1, GSK3β and col1α1, we saw no evidence of EMT, since expression of αSMA and E-cadherin was not changed. Given this gene expression signature was derived from whole lung which may have masked epithelial specific responses, we performed similar experiments using primary cultures of human airway epithelial cells. We show that following exposure to TGFβ1, epithelial cells increase expression of mesenchymal marker EDA-Fn, while down regulating expression of the epithelial marker E-cadherin as expected. Exposure to CSE had no effect on these markers when used alone and did not modify the response to TGFβ1. Recent studies have shown that cigarette smoke condensate (CSC) induces an EMT-like process in BEAS-2B cells [31]. The reasons for these different findings may relate to the type of cell cultures or duration of exposure to CSE, since Veljkovic and colleagues exposed their cells for 30 days, whereas we used 48 h.

## Conclusions

In summary, the main findings of this study was that exposure to CSE in vitro and in vivo induces a profound inflammatory and pro-apoptotic response of the epithelium under normal conditions, but these responses are blunted in COPD-AEC. Furthermore, CSE does not induce an EMT-like phenotype in vitro or in vivo, at least at the time points measured. Our results highlight the capacity of the epithelium to respond to environmental insult is compromised in COPD.

## Additional file

Additional file 1: Figure S1. Cigarette smoke extract (CSE) at concentrations of 50% or lower did not change normal epithelial cell morphology. **Figure S2**. Cigarette smoke extract (CSE) and/or TGFβ1 did not increase cell cytotoxicity in lung epithelial cells. (DOCX 903 kb)
