# Supplementary material for: Acute cigarette smoke exposure activates apoptotic and inflammatory programs but a second stimulus is required to induce epithelial to mesenchymal transition in COPD epithelium
Source: Respir Res. 2017 May 3;18:82. doi: 10.1186/s12931-017-0565-2 (PMC5415733; doi:10.1186/s12931-017-0565-2)
Supplement: Additional file 1: Figure S1. — Cigarette smoke extract (CSE) at concentrations of 50% or lower did not change normal epithelial cell morphology. Figure S2. Cigarette smoke extract (CSE) and/or TGFβ1 did not increase cell cytotoxicity in lung epithelial cells. (DOCX 903 kb) [file 12931_2017_565_MOESM1_ESM.docx]

**Additional file**


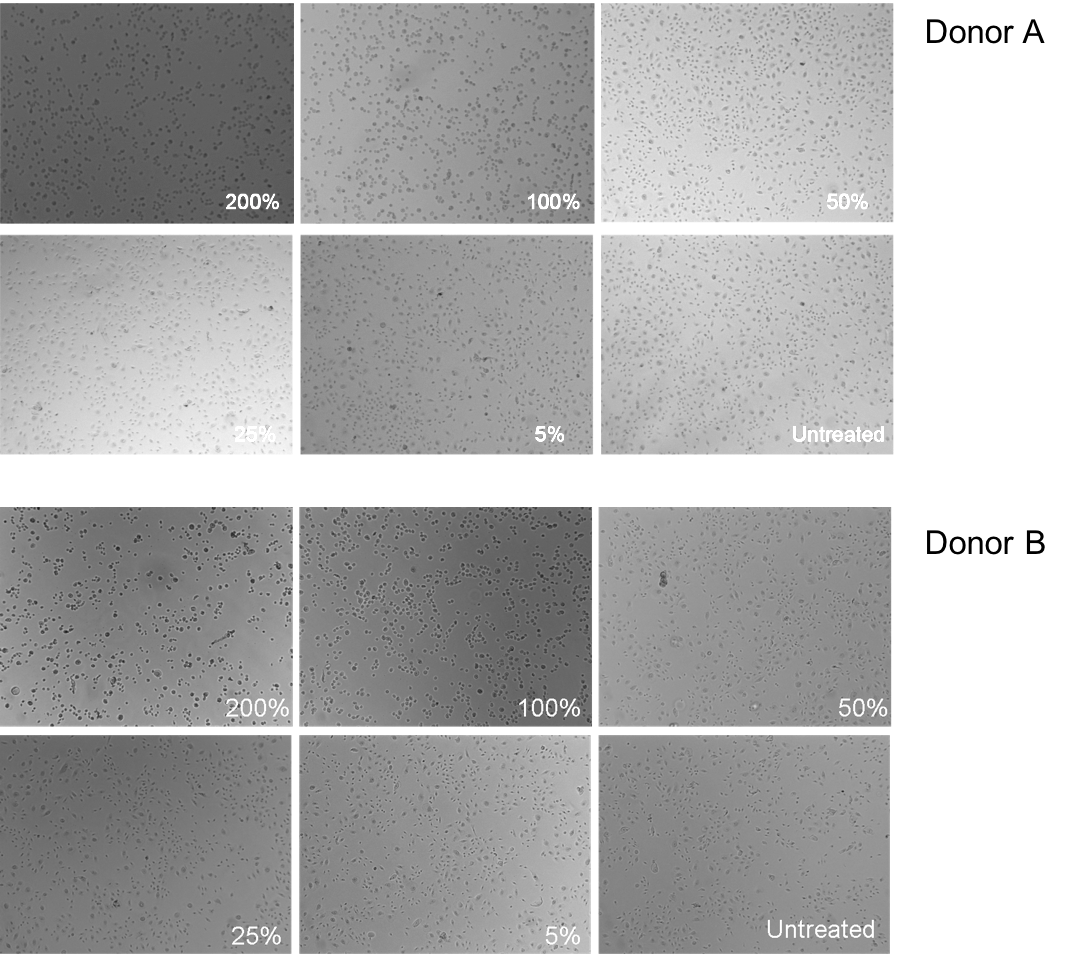


**Additional file 1: Figure S1. Cigarette smoke extract (CSE) at concentrations of 50% or lower did not change normal epithelial cell morphology**. NHBEs (normal human bronchial epithelial cells) from 2 different donors (“Donor A” and “Donor B”) were treated with increasing concentrations of CSE (5-200% as indicated). After 24 hours, phase contrast images were taken and at concentrations of 50% and lower NHBE cells maintained an epithelial morphology (*n*=2).

**A**

**B**

**Additional file 1: Figure S2.** **Cigarette smoke extract (CSE) and/or TGFβ1 did not increase cell cytotoxicity in lung epithelial cells**. NHBEs (normal human bronchial epithelial cells) or COPD-AECs (COPD diseased human bronchial epithelial cells) were treated with increasing concentrations of CSE (5-20% as indicated) and after 24 hours cell cytotoxicity was not further increased compared to untreated (A) as determined by LDH activity measurement (*n*=3). COPD-AECs were treated with CSE (5 and 20%) +/- TGFβ1 (10ng/ml) and after 48 hours there was no additional increase in cell cytotoxicity compared to untreated (B) as assessed by LDH activity measurement (*n*=3).
